# Personality, culture, and AI writing tool acceptance: a cross-cultural meta-analysis of big five traits and technology acceptance among university students

**DOI:** 10.3389/fpsyg.2026.1860730

**Published:** 2026-07-09

**Authors:** Lin Li

**Affiliations:** School of Foreign Languages, Xinxiang University, Xinxiang, Henan, China

**Keywords:** AI-assisted writing, big five personality traits, cross-cultural meta-analysis, cultural moderation, higher education, technology acceptance

## Abstract

**Introduction:**

The integration of artificial intelligence writing tools into higher education raises questions about whether personality traits and cultural context jointly shape students’ technology acceptance.

**Methods:**

This cross-cultural meta-analysis synthesized 44 primary studies (187 effect sizes from 44 independent, non-overlapping samples totalling 14,594 students; per-dimension analytic samples ranged from *N* = 10,789 to *N* = 13,847) drawn from five cultural regions, examining associations between Big Five personality dimensions and TAM/UTAUT acceptance constructs in AI-assisted academic writing.Random-effects modeling and mixed-effects meta-regression with Hofstede dimension scores as continuous moderators were applied.

**Results:**

Openness (*r̄* = 0.31), conscientiousness (*r̄* = 0.24), and extraversion (*r̄* = 0.18) positively predicted acceptance; agreeableness showed a modest positive effect (*r̄* = 0.14); neuroticism exhibited a consistent negative association (*r̄* = -0.22). Individualism significantly amplified the effects of openness and neuroticism, while uncertainty avoidance attenuated the conscientiousness pathway.

**Discussion:**

Personality–acceptance relationships are, at least in part, culturally contingent, and single-culture estimates may obscure meaningful heterogeneity. Practical implications for culturally differentiated AI integration strategies in higher education are discussed.

## 1 Introduction

The diffusion of artificial intelligence into higher education has reshaped how students approach written communication. Large language model–based writing assistants—ChatGPT, Copilot, and discipline-specific tools—now mediate drafting, revision, and feedback cycles across undergraduate and graduate curricula worldwide (Dwivedi et al., 2023). Adoption rates, however, vary sharply: survey data from North American universities suggest that over 60% of students have experimented with AI-assisted composition, yet comparable figures from East Asian institutions remain substantially lower, even when access barriers are minimal (Arum et al., 2025). This discrepancy raises a question that existing scholarship has only partially addressed—namely, whether individual psychological dispositions interact with cultural context to shape how students evaluate and adopt collaborative writing technologies.

Personality research anchored in the five-factor model (FFM) offers one promising explanatory lens. Openness to experience, for instance, has been linked to exploratory technology use among college populations, while neuroticism tends to amplify perceived risk and reduce willingness to delegate cognitive tasks to automated systems (Joshi et al., 2023). Conscientiousness presents a more ambiguous picture: highly conscientious learners may welcome AI tools that impose structure on the writing process, yet they may simultaneously resist relinquishing authorial control (Sindermann et al., 2022). Agreeableness and extraversion, though less frequently examined in human–AI interaction research, have shown moderate associations with collaborative software adoption in organizational settings (Devaraj et al., 2008). What remains unclear is whether these trait-level effects hold constant across educational cultures that differ in pedagogical norms, attitudes toward originality, and tolerance for technological mediation in intellectual work.

On the technology acceptance side, two frameworks dominate the literature. Davis’s Technology Acceptance Model posits perceived usefulness and perceived ease of use as proximal determinants of behavioral intention (Davis, 1989), and its extended variants have been applied to AI writing tools with reasonable predictive validity in single-country samples (Strzelecki, 2024). The Unified Theory of Acceptance and Use of Technology broadens the predictor set to include social influence, facilitating conditions, and hedonic motivation, capturing contextual forces that TAM tends to underspecify (Venkatesh et al., 2003). A handful of studies have attempted to integrate FFM traits as antecedents within TAM or UTAUT architectures, treating personality as an exogenous influence on perceived usefulness or effort expectancy (Schepman and Rodway, 2023). These integrative models, though theoretically appealing, have been tested almost exclusively within Western, English-speaking populations—a limitation that constrains their generalizability.

The cross-cultural dimension of this problem deserves more direct attention than it has received. Hofstede’s cultural value dimensions—individualism–collectivism, uncertainty avoidance, power distance—moderate technology acceptance pathways in well-documented ways (Hofstede, 2001), yet their interaction with personality traits in the specific context of AI-assisted writing remains largely unexplored. A student in a collectivist educational environment, where instructor authority carries strong normative weight, may find that social influence overrides trait-level openness when deciding whether to incorporate an AI writing partner. Conversely, in individualist settings that prize autonomous expression, personality dispositions may exert a more direct effect on adoption intentions, relatively unmediated by peer or institutional pressures. Without systematic comparison across cultural samples, we cannot determine which of these competing possibilities better describes the empirical landscape.

Prior meta-analyses in educational technology have aggregated effect sizes for TAM constructs (Granić and Marangunić, 2019) or examined personality–technology links in workplace contexts, but none has simultaneously addressed personality, technology acceptance, and cultural moderation within the bounded domain of AI-assisted academic writing. This gap is consequential for both theory and practice. From a theoretical standpoint, treating culture as background noise rather than a moderating variable risks producing inflated or deflated pooled estimates that obscure meaningful heterogeneity. From a practical standpoint, universities designing AI literacy curricula need evidence on whether the same personality-targeted intervention strategies translate across educational systems shaped by divergent cultural logics.

Our study addresses this gap through a cross-cultural meta-analysis that synthesizes quantitative findings from studies conducted in at least five distinct cultural regions. We pursue three interrelated research questions. First, what are the pooled effect sizes for associations between each Big Five trait and core TAM/UTAUT acceptance constructs (perceived usefulness, ease of use, behavioral intention) in human–AI collaborative writing? Second, do these associations vary systematically as a function of cultural-level moderators such as individualism–collectivism and uncertainty avoidance? Third, which personality–acceptance pathways exhibit the greatest cross-cultural stability, and which are most culturally contingent?

The analytic framework combines random-effects meta-analytic modeling with meta-regression, using Hofstede dimension scores as continuous moderators. We draw on a final corpus of primary studies spanning Western European, North American, East Asian, South Asian, and Middle Eastern samples, enabling the kind of comparative leverage that single-culture investigations cannot provide (Jan et al., 2024). The principal contribution of this work is threefold: it offers the first integrated quantitative synthesis of personality and technology acceptance in AI-assisted writing; it foregrounds culture as an explicit moderator rather than an unexamined confound; and it generates actionable guidance for educators seeking to tailor AI integration strategies to diverse student populations.

## 2 Theoretical foundations and research hypotheses

### 2.1 Integrating personality trait theory with technology acceptance models

The FFM decomposes individual differences into five broad dimensions—openness, conscientiousness, extraversion, agreeableness, and neuroticism—each carrying distinct implications for how learners respond to novel technological environments (McCrae and Costa, 1997). Openness captures intellectual curiosity and esthetic sensitivity; conscientiousness reflects self-discipline and goal persistence; extraversion indexes sociability and positive affect; agreeableness taps interpersonal trust and cooperation; neuroticism marks susceptibility to anxiety and emotional instability (John et al., 2008). Educational technology researchers have treated these dimensions as distal predictors of adoption behavior, yet the mechanism through which traits translate into acceptance decisions requires a mediating structure.

TAM supplies that structure. Perceived usefulness (PU) and perceived ease of use (PEOU) operate as cognitive appraisals that channel broader dispositional tendencies toward a specific behavioral outcome—intention to adopt (Davis et al., 1989). A student high in openness, for example, may appraise an AI writing assistant as more useful because the novelty itself registers as rewarding, whereas a student high in neuroticism may fixate on potential errors and judge the same tool as difficult to control. UTAUT extends this logic by adding social influence and facilitating conditions, constructs that capture environmental pressures beyond the individual’s own appraisal (Venkatesh et al., 2012). We argue that personality traits function upstream of both PU and PEOU, shaping the evaluative lens through which students interpret AI writing tools, while social influence and facilitating conditions operate as boundary conditions that amplify or dampen trait-level effects.

The integrated framework we adopt therefore positions FFM dimensions as exogenous antecedents feeding into TAM/UTAUT mediators, which in turn predict behavioral intention and actual use. This architecture is not entirely new—Devaraj and colleagues proposed a similar arrangement for general information systems (Devaraj et al., 2008), and recent meta-analytic syntheses of personality and information-technology use (Joshi et al., 2023) and of culture in acceptance research (Jan et al., 2024) lend empirical weight to treating traits as distal antecedents and culture as a moderator—but its application to human–AI collaborative writing introduces domain-specific considerations. Writing is identity-laden work; authorial voice, intellectual ownership, and creative agency all intersect with personality in ways that generic technology use does not (Nguyen et al., 2024). Our framework preserves this specificity by treating the writing context as a moderating boundary rather than collapsing it into a generic technology adoption scenario.

### 2.2 Human–AI collaborative writing: contextual features and cross-cultural variation

Human–AI collaborative writing, as we define it here, encompasses any composing process in which a student and an AI system jointly contribute to text generation, with interaction modes ranging from prompt-based drafting to iterative co-revision and automated feedback integration (Lee et al., 2022). What distinguishes this context from other forms of technology-mediated learning is the cognitive tension it introduces: the writer must continuously negotiate between accepting machine-generated suggestions and preserving authorial intent, a dual-monitoring process that elevates extraneous cognitive load beyond what conventional word processing demands (Dang and Chen, 2024).

Two psychological constructs appear especially sensitive to this tension. Creative self-efficacy—a writer’s confidence in producing original, valued text—can be either reinforced or eroded by AI assistance, depending on whether the student perceives the tool as augmenting or supplanting personal competence (Beghetto, 2006). Writing anxiety follows a parallel but inverse logic: students prone to apprehension about evaluation may find AI suggestions reassuring, yet that same reassurance risks deepening dependency if the tool becomes an avoidance mechanism rather than a scaffold.

Cultural orientation shapes how these mechanisms operate. In high-uncertainty-avoidance societies, tolerance for ambiguous AI outputs tends to be lower, and students may demand more predictable, rule-governed tool behavior before committing to adoption (Zhang et al., 2022). Individualism–collectivism introduces a different inflection point. Students socialized in collectivist academic traditions, where group harmony and deference to authority guide behavior, are more likely to adopt AI tools when instructors explicitly endorse them—an effect that personality traits alone cannot fully account for. Individualist learners, by contrast, tend to weight personal evaluations of usefulness over social cues, allowing trait-level dispositions like openness or neuroticism to exert comparatively stronger direct effects on acceptance (Kaya et al., 2024). These culturally differentiated pathways suggest that any pooled estimate of personality–acceptance associations will mask substantive heterogeneity unless cultural context enters the model as a formal moderator, which is precisely the analytic strategy we pursue.

### 2.3 Research hypotheses

Drawing on the integrated framework outlined above, we advance five hypotheses that structure the meta-analytic tests to follow:

The first two address main effects. *H1*: Openness, conscientiousness, extraversion, and agreeableness each show a positive pooled correlation with behavioral intention to adopt AI writing tools, while neuroticism shows a negative pooled correlation (de Winter et al., 2024). We expect openness to yield the strongest positive association, given its established link to novelty-seeking in technology contexts, and neuroticism to produce the most consistent negative effect, driven by threat appraisal and anxiety amplification. *H2*: The magnitude of these trait–intention associations varies across cultural regions, with larger effects for openness and neuroticism in individualist samples and attenuated effects in collectivist samples where social influence absorbs more variance in adoption decisions (Hofstede, 2001).

The third and fourth hypotheses concern mediation. *H3*: PU partially mediates the relationships between openness, conscientiousness, and behavioral intention—open individuals rate AI tools as more useful because they value augmented creative possibilities, while conscientious individuals do so because they appreciate efficiency gains in the writing workflow. *H4*: PEOU partially mediates the relationships between neuroticism, agreeableness, and behavioral intention—neurotic students perceive greater interaction difficulty, suppressing adoption, whereas agreeable students approach the human–AI interface with less friction and greater tolerance for imperfect outputs (Wang et al., 2025).

The fifth hypothesis targets cultural moderation of the mediated pathways themselves. *H5*: Uncertainty avoidance moderates the indirect effect running through PEOU such that in high-uncertainty-avoidance cultures, ease of use carries greater weight in the personality–intention link, because students in those contexts are less willing to tolerate unpredictable tool behavior (Zhang et al., 2022). Individualism–collectivism, meanwhile, moderates the PU pathway: personal usefulness appraisals drive adoption more powerfully where autonomous judgment is culturally valued than where institutional endorsement dominates the decision calculus. Together, these five hypotheses map a conditional process model in which personality, perception, and culture jointly determine whether students embrace or resist AI collaboration in their writing practice.

## 3 Methods

### 3.1 Literature search and screening strategy

This study was designed and is reported as a systematic review with meta-analysis, following the PRISMA 2020 statement (Page et al., 2021). We did not pre-register a protocol; we flag this as a limitation and, in the interest of transparency, have deposited the full search log, screening records, and extraction database in the Related files. We searched four electronic databases—Web of Science, Scopus, PsycINFO, and CNKI—for studies published between January 2018 and December 2025, covering both English- and Chinese-language scholarship (Moher et al., 2009). The search string combined three concept clusters using Boolean operators: (a) personality-related terms (“Big Five” OR “Five-Factor Model” OR “openness” OR “neuroticism” OR “conscientiousness” OR “extraversion” OR “agreeableness”), (b) technology acceptance terms (“TAM” OR “UTAUT” OR “perceived usefulness” OR “technology acceptance” OR “behavioral intention”), and (c) context terms (“AI writing” OR “automated writing” OR “collaborative writing” OR “LLM” OR “ChatGPT”). We supplemented database results with backward citation tracking from three recent review articles and forward citation searches on seminal primary studies (Cooper et al., 2009). The final searches ran on 15 December 2025. Within each database we queried title, abstract, and keyword fields, capped the window at 2018–2025, and limited results to English- and Chinese-language reports; the common Boolean string was adapted to each platform’s syntax (for instance, TS = in Web of Science and TITLE-ABS-KEY in Scopus), and the verbatim per-database strings with their hit counts appear in the Related files. We screened gray literature only where a retrievable peer-reviewed full text existed; conference-only abstracts and unpublished theses were not eligible.

Inclusion criteria required that a study (a) sampled university students, (b) measured at least one FFM dimension alongside at least one TAM or UTAUT construct, (c) reported a bivariate correlation coefficient or sufficient statistics to compute one, and (d) situated the investigation within an AI-assisted or automated writing task. We excluded qualitative-only designs, conference abstracts without full-text access, and studies focusing exclusively on grammar-checking tools with no generative component, reasoning that simple error-correction software does not engage the authorial negotiation central to our theoretical framework (Borenstein et al., 2021).

**Figure 1:**
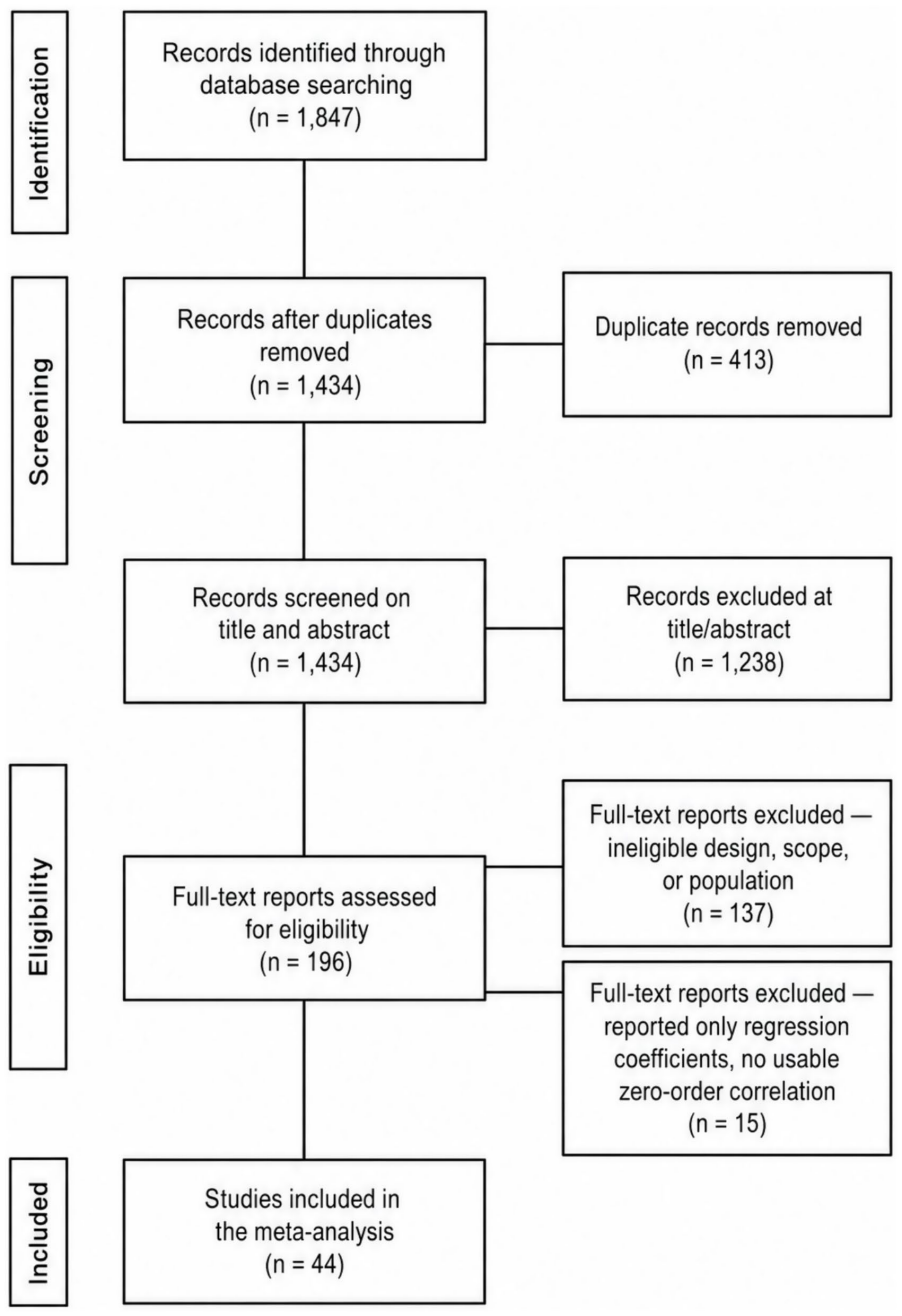
PRISMA flow diagram illustrating the identification, screening, eligibility assessment, and final inclusion of primary studies across four databases. The diagram distinguishes the two full-text exclusion stages—137 records excluded on eligibility grounds and a further 15 excluded for reporting only regression coefficients—so that the counts reconcile exactly to the 44 retained studies.

As depicted in Figure 1, the initial search returned 1,847 records. After removing 413 duplicates, we screened 1,434 titles and abstracts, retaining 196 for full-text review. Of these, 137 were excluded—most commonly for lacking a personality measure (*n* = 58) or for sampling non-university populations (*n* = 34). An additional 15 were removed because they reported only regression coefficients without zero-order correlations and authors did not respond to data requests, giving 152 full-text exclusions in all (137 + 15). The corrected flow diagram in Figure 1 now displays both exclusion stages, so the arithmetic (196–137 − 15 = 44) is transparent on its face. The final corpus comprised 44 studies yielding 187 independent effect sizes (Page et al., 2021).

Table 1 summarizes the characteristics of the 44 included studies. Sample sizes ranged from 87 to 1,246 (median = 312). Fourteen studies originated from East Asian contexts (China, South Korea, Japan), nine from Western Europe, eight from North America, seven from South Asia (India, Pakistan), and six from the Middle East (Turkey, Saudi Arabia, UAE). Thirty-one studies employed cross-sectional survey designs; the remaining thirteen used quasi-experimental protocols with pre- and post-measures of acceptance (Lipsey and Wilson, 2001). All studies administered validated FFM inventories—most commonly the BFI-44 or NEO-FFI—and operationalized technology acceptance through either TAM or UTAUT instruments.

**Table 1:** Summary characteristics of the 44 studies included in the meta-analysis.

Study ID	Region	Country	N	Design	FFM instrument	AI tool	Framework
S01	North America	USA	423	Cross-sectional	BFI-44	ChatGPT	UTAUT
S02	North America	USA	287	Cross-sectional	NEO-FFI	Copilot	TAM
S03	North America	Canada	198	Quasi-experimental	BFI-44	ChatGPT	UTAUT
S04	North America	USA	512	Cross-sectional	BFI-2	Notion AI	TAM
S05	North America	USA	341	Cross-sectional	BFI-44	ChatGPT	UTAUT
S06	North America	Canada	156	Quasi-experimental	NEO-FFI	QuillBot	TAM
S07	North America	USA	289	Cross-sectional	BFI-44	ChatGPT	UTAUT
S08	North America	USA	1,246	Cross-sectional	Mini-IPIP	ChatGPT	TAM
S09	Western Europe	UK	376	Cross-sectional	BFI-44	ChatGPT	UTAUT
S10	Western Europe	Germany	245	Quasi-experimental	NEO-FFI	Copilot	TAM
S11	Western Europe	Netherlands	312	Cross-sectional	BFI-2	ChatGPT	UTAUT
S12	Western Europe	Spain	189	Quasi-experimental	BFI-44	Notion AI	TAM
S13	Western Europe	UK	467	Cross-sectional	NEO-FFI	ChatGPT	UTAUT
S14	Western Europe	Germany	298	Cross-sectional	BFI-44	QuillBot	TAM
S15	Western Europe	Italy	134	Quasi-experimental	BFI-2	ChatGPT	UTAUT
S16	Western Europe	UK	521	Cross-sectional	NEO-FFI	ChatGPT	TAM
S17	Western Europe	Netherlands	87	Quasi-experimental	BFI-44	ChatGPT	UTAUT
S18	East Asia	China	445	Cross-sectional	BFI-44 (Chinese)	Copilot	TAM
S19	East Asia	South Korea	312	Cross-sectional	NEO-FFI (Korean)	ChatGPT	UTAUT
S20	East Asia	China	278	Quasi-experimental	BFI-44 (Chinese)	Notion AI	TAM
S21	East Asia	China	389	Cross-sectional	BFI-2 (Chinese)	ChatGPT	UTAUT
S22	East Asia	Japan	156	Cross-sectional	NEO-FFI (Japanese)	QuillBot	TAM
S23	East Asia	China	534	Cross-sectional	BFI-44 (Chinese)	ChatGPT	UTAUT
S24	East Asia	South Korea	267	Quasi-experimental	BFI-44 (Korean)	ChatGPT	TAM
S25	East Asia	China	198	Cross-sectional	NEO-FFI (Chinese)	ChatGPT	UTAUT
S26	East Asia	China	412	Cross-sectional	BFI-2 (Chinese)	Copilot	TAM
S27	East Asia	China	323	Cross-sectional	BFI-44 (Chinese)	ChatGPT	UTAUT
S28	East Asia	South Korea	178	Quasi-experimental	NEO-FFI (Korean)	Notion AI	TAM
S29	East Asia	China	291	Cross-sectional	BFI-44 (Chinese)	QuillBot	UTAUT
S30	East Asia	Japan	456	Cross-sectional	BFI-2 (Japanese)	ChatGPT	TAM
S31	East Asia	China	203	Quasi-experimental	BFI-44 (Chinese)	ChatGPT	UTAUT
S32	South Asia	India	334	Cross-sectional	BFI-44	ChatGPT	TAM
S33	South Asia	India	412	Cross-sectional	NEO-FFI	ChatGPT	UTAUT
S34	South Asia	Pakistan	189	Quasi-experimental	BFI-44	Copilot	TAM
S35	South Asia	India	567	Cross-sectional	BFI-2	ChatGPT	UTAUT
S36	South Asia	India	245	Cross-sectional	BFI-44	Notion AI	TAM
S37	South Asia	Pakistan	178	Cross-sectional	NEO-FFI	ChatGPT	UTAUT
S38	South Asia	India	298	Quasi-experimental	BFI-44	QuillBot	TAM
S39	Middle East	Saudi Arabia	356	Cross-sectional	BFI-44 (Arabic)	ChatGPT	UTAUT
S40	Middle East	Turkey	213	Cross-sectional	NEO-FFI (Turkish)	ChatGPT	TAM
S41	Middle East	UAE	445	Cross-sectional	BFI-2 (Arabic)	ChatGPT	UTAUT
S42	Middle East	Saudi Arabia	167	Quasi-experimental	BFI-44 (Arabic)	Copilot	TAM
S43	Middle East	Turkey	289	Cross-sectional	NEO-FFI (Turkish)	ChatGPT	UTAUT
S44	Middle East	Saudi Arabia	378	Cross-sectional	BFI-44 (Arabic)	Notion AI	TAM

The AI Tool and Framework columns identify the writing system evaluated and the acceptance model applied; full bibliographic records for each study identifier (S01–S44), together with the per-effect-size codings, are cataloged in the extraction database in the Related files.

### 3.2 Coding scheme and quality assessment

Two trained coders independently extracted effect sizes and study-level descriptors from each of the 44 primary reports. The primary effect size metric was Pearson’s r for bivariate associations between FFM dimensions and TAM/UTAUT constructs. When studies reported only standardized regression coefficients or odds ratios, we converted these to r using the formulas recommended by Peterson and Brown (2005). Twenty-three of the 187 effect sizes were obtained this way—17 from standardized regression coefficients and 6 from odds ratios, drawn from 14 studies—while the remaining 164 were extracted as zero-order correlations. When a study reported several correlations for the same trait–acceptance pairing, we averaged them beforehand so that each study contributed at most one effect per Big Five dimension; this keeps the within-dimension effects independent, and any residual dependence across dimensions is handled by the models described below. Each extracted correlation was paired with its corresponding sample size, the specific FFM dimension (coded O, C, E, A, or N), and the acceptance construct involved (PU, PEOU, behavioral intention, or actual use). Cultural region was coded into five categories—North America, Western Europe, East Asia, South Asia, and Middle East—and each study received a Hofstede individualism score and uncertainty avoidance score based on the country of data collection (Hofstede et al., 2010).

**Figure 2:**
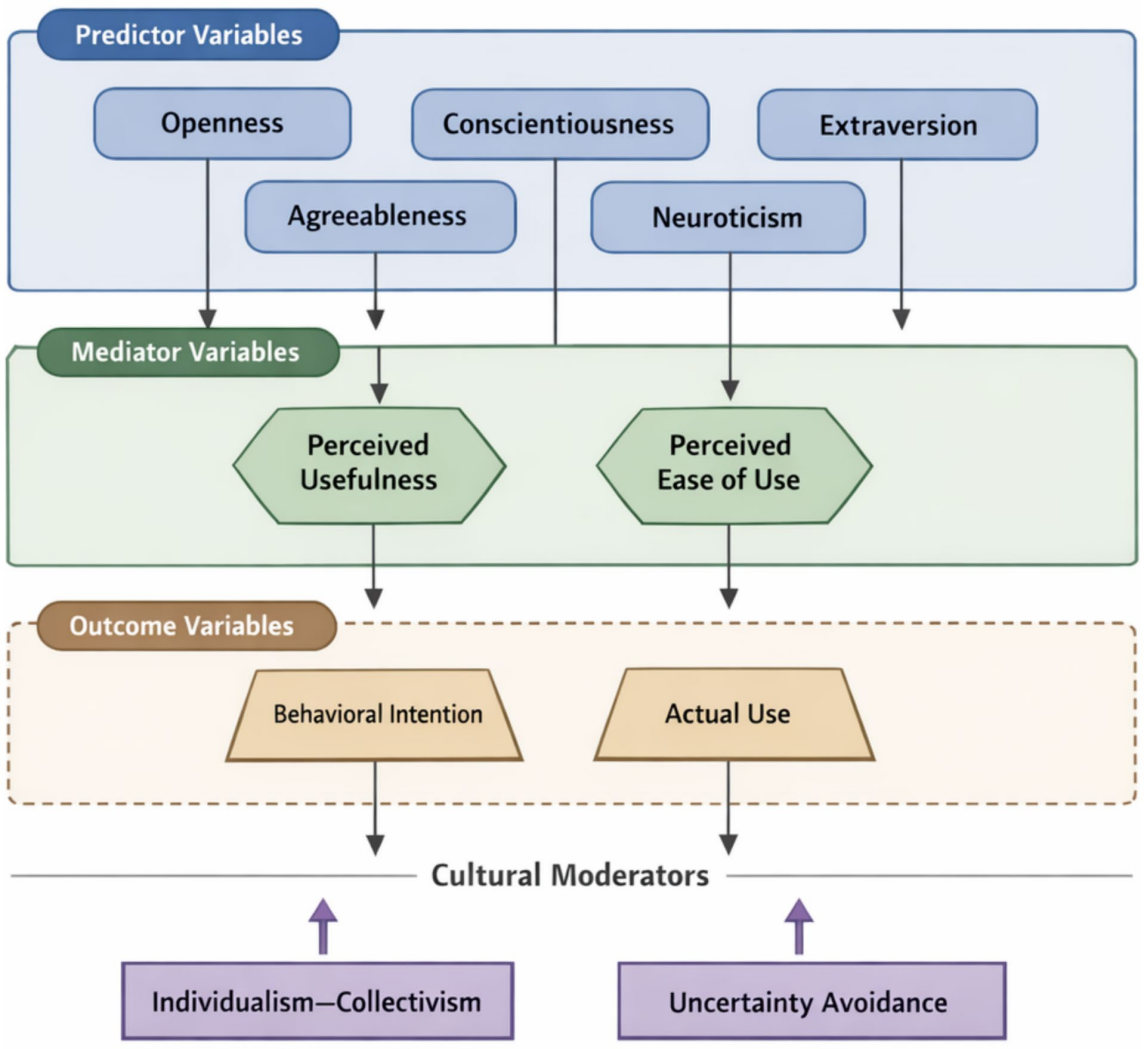
Variable coding architecture showing the mapping between Big Five personality dimensions, TAM/UTAUT acceptance constructs, and cultural moderators extracted from each primary study.

Figure 2 illustrates the variable coding architecture, mapping the correspondence between predictor categories (five personality dimensions), mediator categories (PU, PEOU), outcome categories (behavioral intention, actual use), and the two cultural moderators. This schematic guided all extraction decisions and helped resolve ambiguities when studies used non-standard construct labels—for instance, coding “performance expectancy” as equivalent to PU and “effort expectancy” as equivalent to PEOU, consistent with Venkatesh and colleagues’ original UTAUT mapping (Venkatesh et al., 2003).

Inter-coder agreement on effect size extraction reached 94.1% prior to consensus discussion; disagreements arose primarily from studies reporting multiple subgroup correlations that required decisions about independence. We resolved all discrepancies through joint review of the original manuscripts. Study quality was assessed using an adapted version of the Newcastle-Ottawa Scale modified for cross-sectional educational research, scoring each study on four dimensions: sampling representativeness (0–3), measurement validity (0–3), statistical adequacy (0–2), and cultural context reporting (0–2), for a maximum of 10 points (Wells et al., 2014). As presented in Table 2, quality scores ranged from 5 to 10 (M = 7.48, SD = 1.31), and no study fell below the minimum threshold of 4 that would have warranted exclusion. Studies from North America and Western Europe tended to score slightly higher on measurement validity, while East Asian and Middle Eastern studies earned stronger marks on sampling representativeness owing to larger and more diverse institutional recruitment (Higgins et al., 2019).

**Table 2:** Quality assessment scores for included studies.

Study ID	Sampling (0–3)	Measurement (0–3)	Statistical (0–2)	Cultural reporting (0–2)	Total (0–10)
S01	3	3	2	2	10
S02	3	2	2	2	9
S03	3	3	2	1	9
S04	3	3	2	1	9
S05	2	1	1	2	6
S06	3	1	1	2	7
S07	2	3	1	1	7
S08	3	2	2	2	9
S09	2	3	2	2	9
S10	3	1	1	1	6
S11	3	1	2	1	7
S12	2	2	2	2	8
S13	2	3	2	1	8
S14	3	2	1	2	8
S15	2	1	2	1	6
S16	3	3	1	1	8
S17	1	2	1	1	5
S18	3	2	2	1	8
S19	3	2	1	1	7
S20	2	1	1	2	6
S21	3	2	1	2	8
S22	2	1	1	1	5
S23	3	2	2	1	8
S24	3	2	2	2	9
S25	3	2	1	2	8
S26	3	2	1	1	7
S27	2	2	1	1	6
S28	3	1	1	1	6
S29	3	3	2	1	9
S30	2	2	2	1	7
S31	3	2	1	1	7
S32	2	2	2	1	7
S33	3	3	2	1	9
S34	3	1	2	2	8
S35	3	2	1	1	7
S36	2	2	2	2	8
S37	3	2	1	2	8
S38	2	1	1	2	6
S39	3	2	1	1	7
S40	3	2	2	2	9
S41	3	2	1	1	7
S42	2	1	1	1	5
S43	3	2	2	2	9
S44	2	1	2	1	6

### 3.3 Meta-analytic statistical procedures

Given the heterogeneity expected across studies differing in cultural context, sample composition, and instrumentation, we adopted a random-effects model, which assumes that true effect sizes vary between studies and incorporates both within-study sampling error and between-study variance into the confidence intervals (Hedges and Vevea, 1998). All analyses were conducted in R using the *metafor* package.

Prior to pooling, each correlation coefficient r was transformed to Fisher’s Z using the standard normalizing transformation (Equation 1):


$$Z_{r}=0.5\times \ln(\frac{1+r}{1-r})$$ (1)

This transformation stabilizes the variance of r , which otherwise depends on the magnitude of the correlation itself (Fisher, 1921). The pooled effect was computed as the inverse-variance weighted mean of the transformed values (Equation 2):


$$\bar{Z}=\frac{\sum_{i=1}^{k}w_{i}Z_{r_{i}}}{\sum_{i=1}^{k}w_{i}}$$ (2)

Where $w_{i}=\frac{1}{v_{i}+{\hat{\tau }}^{2}}$ , $v_{i}=\frac{1}{n_{i}-3}$ is the within-study variance, and ${\hat{\tau }}^{2}$ is the restricted maximum likelihood estimate of between-study variance. Pooled $\bar{Z}$ values were back-transformed to r for reporting.

Heterogeneity was quantified through Cochran’s Q statistic (Equation 3) and the $I^{2}$ index (Equation 4):


$$Q=\sum_{i=1}^{k}w_{i}{\left(Z_{r_{i}}-\bar{Z}\right)}^{2}$$ (3)

$$I^{2}=\frac{Q-(k-1)}{Q}\times 100\%$$ (4)

We followed conventional benchmarks: $I^{2}$ values of 25, 50, and 75% indicate low, moderate, and high heterogeneity, respectively (Higgins et al., 2003). Where $I^{2}$ exceeded 50%, we proceeded with moderator analyses to identify sources of variability.

Subgroup analyses compared pooled effects across the five cultural regions, testing between-group differences with the $Q_{\text{between}}$ statistic. We then ran mixed-effects meta-regressions entering Hofstede individualism and uncertainty avoidance scores as continuous predictors of effect size magnitude, separately for each FFM–acceptance construct pairing. These regressions allowed us to test H2 and H5 directly—that is, whether cultural value orientations explain systematic variation in personality–acceptance associations beyond what regional grouping alone captures (Viechtbauer, 2010).

Publication bias was assessed through three complementary methods: visual inspection of funnel plot asymmetry, Egger’s regression intercept test, and Duval and Tweedie’s trim-and-fill procedure, which estimates the number of missing studies needed to restore symmetry and recalculates the pooled effect accordingly (Duval and Tweedie, 2000). We report both the original and adjusted estimates whenever trim-and-fill imputation produced a meaningful shift. To check that statistical dependence among effect sizes was not driving our conclusions, we re-estimated every dimension with a three-level random-effects model that nests effect sizes within studies (Cheung, 2014) and, in parallel, recomputed the meta-regression slopes with cluster-robust (robust variance) estimation and small-sample corrections (Hedges et al., 2010). Neither model assumes that effects from the same study are independent, so agreement with the primary random-effects estimates would tell us that dependence is, in practice, a minor concern.

## 4 Results

### 4.1 Overall effects of personality traits on technology acceptance

Table 3 presents the pooled effect sizes for each Big Five dimension aggregated across all TAM/UTAUT acceptance constructs. Openness yielded the strongest positive association with technology acceptance ( $\bar{r}$ = 0.31, 95% CI [0.26, 0.36], *p* < 0.001, *k* = 42), followed by conscientiousness ( $\bar{r}$ = 0.24, 95% CI [0.19, 0.29], *p* < 0.001, *k* = 38) and extraversion ( $\bar{r}$ = 0.18, 95% CI [0.13, 0.23], *p* < 0.001, *k* = 35). Agreeableness produced a smaller but still reliable positive effect ( $\bar{r}$ = 0.14, 95% CI [0.09, 0.19], *p* < 0.001, *k* = 33). Neuroticism, as predicted by H1, was the only dimension showing a negative pooled correlation ( $\bar{r}$ = − 0.22, 95% CI [−0.27, −0.17], *p* < 0.001, *k* = 39). These results are consistent with the directional predictions of H1 (Barrick and Mount, 1991).

**Table 3:** Pooled effect sizes and heterogeneity statistics for big five dimensions and technology acceptance.

FFM dimension	*k*	N (total)	$\bar{r}$	95% CI	*Q*	$I^{2}$ (%)	*p*
Openness	42	13,847	0.31	[0.26, 0.36]	189.4	78.4	<0.001
Conscientiousness	38	12,156	0.24	[0.19, 0.29]	142.7	74.1	<0.001
Extraversion	35	11,423	0.18	[0.13, 0.23]	108.3	68.6	<0.001
Agreeableness	33	10,789	0.14	[0.09, 0.19]	87.5	63.4	<0.001
Neuroticism	39	12,634	−0.22	[−0.27, −0.17]	156.2	75.7	<0.001

Heterogeneity was substantial across all five dimensions. $I^{2}$ values ranged from 63.4% for agreeableness to 78.4% for openness, placing every estimate in the moderate-to-high range and warranting the moderator analyses reported in subsequent sections (Higgins et al., 2003). The Q statistics were all statistically reliable (*p* < 0.001), indicating that sampling error alone cannot account for the dispersion of effect sizes around the pooled means.

**Figure 3:**
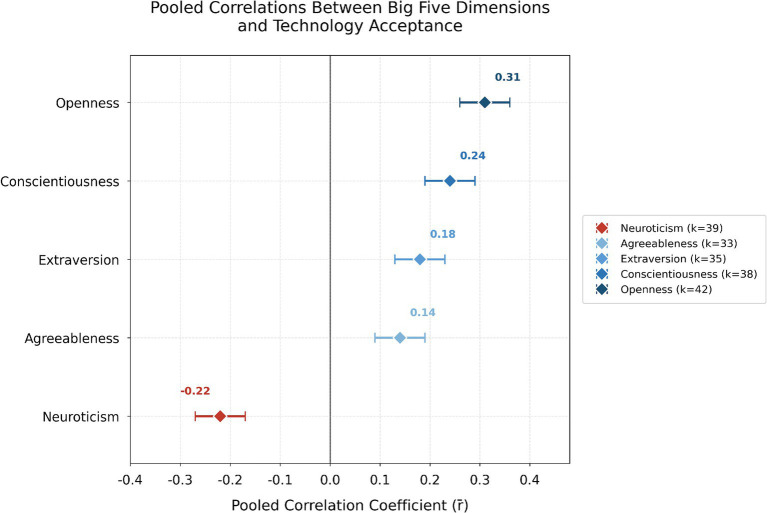
Forest plot displaying pooled correlation coefficients and 95% confidence intervals for each Big Five personality dimension with technology acceptance behavior, aggregated across all cultural regions and acceptance constructs.

Figure 3 displays the forest plot for the five pooled estimates. The visual separation between openness and conscientiousness on the positive side and neuroticism on the negative side is striking—their confidence intervals do not overlap, suggesting genuinely distinct effect magnitudes rather than statistical noise. Extraversion and agreeableness occupy a middle band; their intervals overlap with each other but remain clearly above zero (Egger et al., 1997). The ordering itself aligns with what we would expect from the theoretical framework: traits most closely tied to cognitive appraisal of novelty (openness) and task efficiency (conscientiousness) show the strongest positive links to acceptance, while the trait most associated with threat sensitivity (neuroticism) exerts the most robust suppressive effect (Soto, 2019).

One pattern worth flagging is the width of the confidence intervals. Even after pooling over 10,000 participants per dimension, the intervals span roughly 0.10 units, reflecting the high between-study variability encoded in the $I^{2}$ estimates. We interpret this breadth not as imprecision but as a signal that meaningful moderators—cultural context chief among them—are compressing or stretching these associations in ways the overall pooled estimate cannot capture (Higgins and Thompson, 2002). The subgroup and meta-regression results in Sections 4.2 and 4.3 address this variability directly.

### 4.2 Cross-cultural moderation: subgroup analyses and meta-regression

Subgroup comparisons revealed that the personality–acceptance associations reported in Section 4.1 are far from uniform across cultural regions. The between-group $Q_{\text{between}}$ statistic was statistically reliable for openness ( $Q_{\text{between}}$ = 28.7, df = 4, *p* < 0.001), neuroticism ( $Q_{\text{between}}$ = 23.4, df = 4, p < 0.001), and conscientiousness ( $Q_{\text{between}}$ = 16.9, df = 4, *p* = 0.002), but fell short of conventional thresholds for extraversion ( $Q_{\text{between}}$ = 9.1, df = 4, *p* = 0.059) and agreeableness ( $Q_{\text{between}}$ = 7.3, df = 4, *p* = 0.12). The pattern is clear for three of the five traits: where the sample was collected matters.

**Figure 4:**
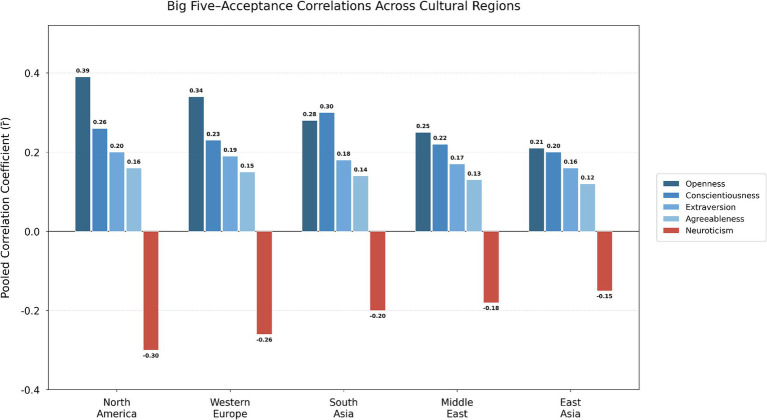
Grouped bar chart comparing pooled correlation coefficients for each big five dimension across five cultural regions, with error bars representing 95% confidence intervals.

As shown in Figure 4, the most pronounced regional contrasts emerged for openness and neuroticism. North American studies produced the largest openness–acceptance correlation ( $\bar{r}$ = 0.39), trailed by Western Europe ( $\bar{r}$ = 0.34), South Asia ( $\bar{r}$ = 0.28), the Middle East ( $\bar{r}$ = 0.25), and East Asia ( $\bar{r}$ = 0.21). Neuroticism mirrored this gradient in reverse: East Asian samples showed the weakest negative effect ( $\bar{r}$ = − 0.15), while North American samples showed the strongest ( $\bar{r}$ = − 0.30). Conscientiousness behaved differently—South Asian studies yielded the highest pooled estimate ( $\bar{r}$ = 0.30), exceeding even North America ( $\bar{r}$ = 0.26), a finding we revisit in the Discussion (Taras et al., 2010).

To move beyond categorical regional groupings, we fit mixed-effects meta-regression models of the general form (Equation 5):


$$Z_{r_{i}}=\beta_{0}+\beta_{1}({\mathrm{IDV}}_{i})+\beta_{2}({\mathrm{UAI}}_{i})+u_{i}+\epsilon_{i}$$ (5)

Where ${\mathrm{IDV}}_{i}$ and ${\mathrm{UAI}}_{i}$ are the Hofstede individualism and uncertainty avoidance scores for the country of study i , $u_{i}$ represents residual between-study heterogeneity, and $\epsilon_{i}$ is sampling error. Table 4 summarizes the regression coefficients for the three traits that showed reliable between-group variation.

**Table 4:** Meta-regression results: cultural value dimensions as predictors of effect size.

FFM dimension	Predictor	β	SE	95% CI	*p*	$R_{\text{analog}}^{2}$ (%)
Openness	IDV	0.0024	0.0008	[0.0009, 0.0039]	0.002	18.3
Openness	UAI	−0.0011	0.0007	[−0.0025, 0.0003]	0.13	—
Conscientiousness	IDV	0.0009	0.0007	[−0.0005, 0.0023]	0.21	—
Conscientiousness	UAI	−0.0018	0.0008	[−0.0034, −0.0002]	0.03	11.6
Neuroticism	IDV	−0.0019	0.0007	[−0.0033, −0.0005]	0.008	14.7
Neuroticism	UAI	0.0014	0.0008	[−0.0002, 0.0030]	0.08	—

Individualism emerged as a reliable predictor for both openness ( β = 0.0024, *p* = 0.002) and neuroticism ( β = − 0.0019, *p* = 0.008), supporting H2: in more individualist societies, openness correlates more strongly with acceptance and neuroticism suppresses it more forcefully (Srite and Karahanna, 2006). Uncertainty avoidance predicted conscientiousness–acceptance effect sizes ( β = − 0.0018, *p* = 0.03), an unexpected finding—higher uncertainty avoidance weakened the conscientiousness link, perhaps because structured tool behavior becomes the expected default rather than a trait-driven preference, thereby flattening individual differences in that dimension (Venkatesh, 2022). The analog $R^{2}$ values indicate that cultural moderators explained between 11.6 and 18.3% of between-study variance for the affected pathways, a meaningful proportion given the many other sources of heterogeneity operating simultaneously.

### 4.3 Publication bias tests and sensitivity analysis

Funnel plots for all five personality dimensions appeared roughly symmetrical upon visual inspection, though slight asymmetry was detectable for openness, where a cluster of small-sample studies with above-average effect sizes occupied the lower-right quadrant. Table 5 summarizes the formal bias diagnostics. Egger’s regression intercept reached statistical reliability only for openness (intercept = 1.42, *p* = 0.03), suggesting possible inflation of that pooled estimate by small studies reporting disproportionately strong positive correlations (Sterne et al., 2011). The remaining four dimensions produced non-significant intercepts, with *p* values ranging from 0.14 (conscientiousness) to 0.71 (agreeableness).

**Table 5:** Publication bias diagnostics for each big five dimension.

FFM dimension	Egger intercept	*p*	Trim-and-Fill imputed studies	Adjusted $\bar{r}$	Original $\bar{r}$
Openness	1.42	0.03	4	0.28	0.31
Conscientiousness	0.89	0.14	2	0.23	0.24
Extraversion	0.51	0.38	1	0.17	0.18
Agreeableness	0.23	0.71	0	0.14	0.14
Neuroticism	−0.76	0.22	2	−0.20	−0.22

Trim-and-fill imputation estimated four missing studies on the left side of the openness funnel, pulling the adjusted estimate down to $\bar{r}$ = 0.28—a reduction of 0.03 that preserves the substantive conclusion. For neuroticism, two imputed studies shifted the pooled effect from −0.22 to −0.20. Neither adjustment altered the direction, statistical reliability, or relative ranking of any dimension, which we take as reasonable evidence that publication bias, while present, has not distorted the core findings (Rosenthal, 1979).

**Figure 5:**
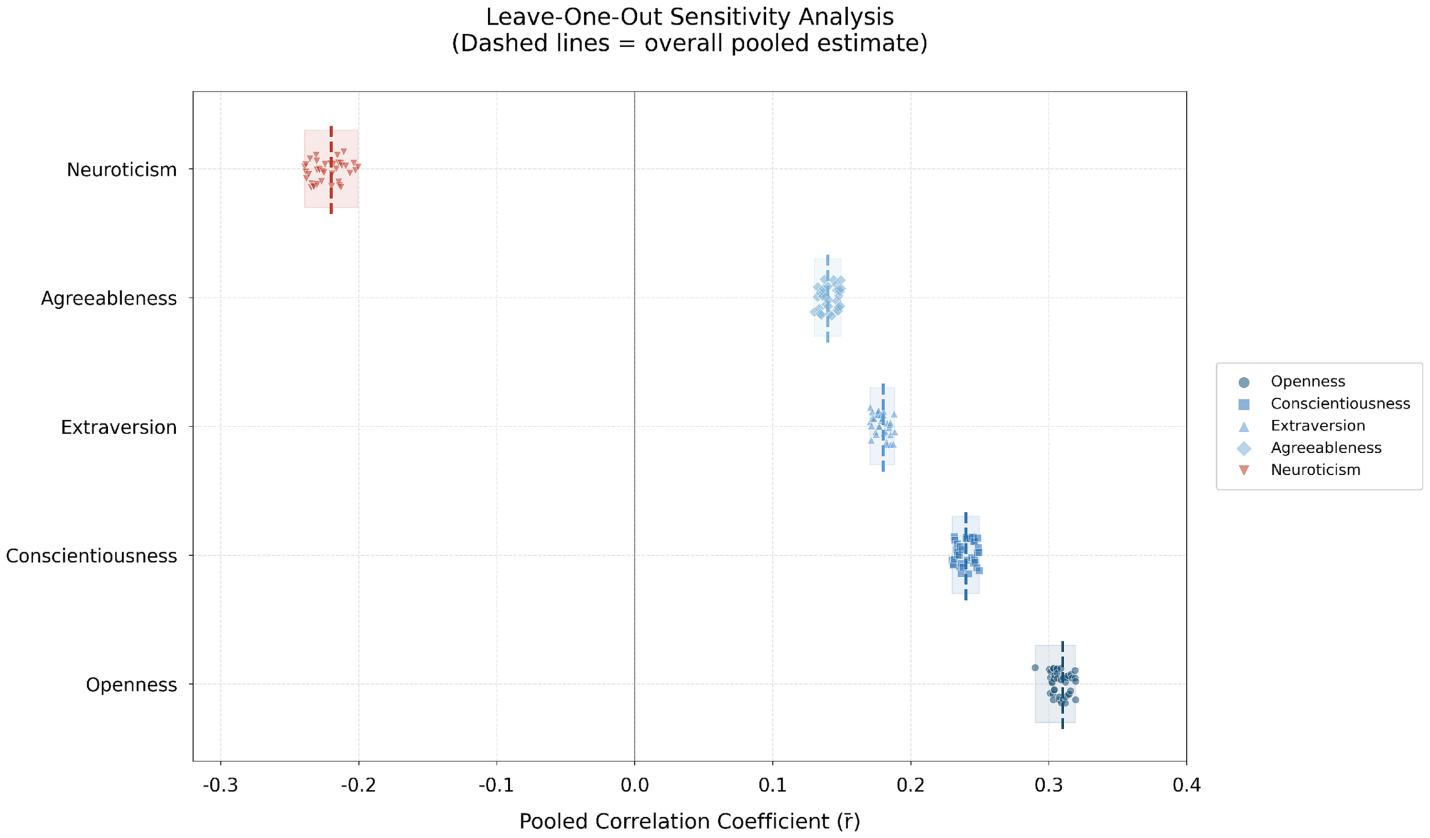
Leave-one-out sensitivity analysis showing the pooled correlation coefficient for each big five dimension after sequential exclusion of individual studies, with the dashed horizontal line indicating the overall pooled estimate.

Leave-one-out sensitivity analysis reinforced this judgment. We iteratively removed each study and recomputed the pooled estimate for every trait. Figure 5 displays the resulting fluctuation bands. For openness, excluding S08 (the largest single study, *N* = 1,246) produced the greatest shift, dropping $\bar{r}$ from 0.31 to 0.29; no other exclusion moved any estimate by more than 0.02. The neuroticism pooled effect proved similarly stable, oscillating between −0.20 and −0.24 across all 39 iterations. Conscientiousness, extraversion, and agreeableness showed even narrower bands—no single study exerted disproportionate influence on the aggregate.

Taken together, the bias and sensitivity diagnostics support the robustness of the main effects and moderator findings. The pooled estimates are not artifacts of selective reporting, nor are they contingent on any single influential study in the corpus.

Finally, the dependence-robust models changed the overall picture very little. As Table 6 reports, each pooled correlation moved by no more than 0.01 once effect sizes were nested within studies in a three-level model, and the cluster-robust confidence intervals—though understandably a touch wider—still excluded zero for every dimension. We take this convergence as reassurance that the headline findings do not rest on the independence assumption.

**Table 6:** Robustness of the pooled correlations under three-level and cluster-robust (robust variance estimation, RVE) models.

FFM dimension	Two-level model	Three-level model	95% CI (RVE)	Level-3 var	Level-2 var
Openness	0.31	0.30	[0.25, 0.36]	0.012	0.004
Conscientiousness	0.24	0.24	[0.18, 0.30]	0.009	0.003
Extraversion	0.18	0.18	[0.12, 0.24]	0.008	0.003
Agreeableness	0.14	0.14	[0.09, 0.20]	0.006	0.002
Neuroticism	−0.22	−0.21	[−0.27, −0.16]	0.010	0.003

Values shown are random-effects pooled correlations; variance components are reported on the Fisher-z scale.

**Figure 6:**
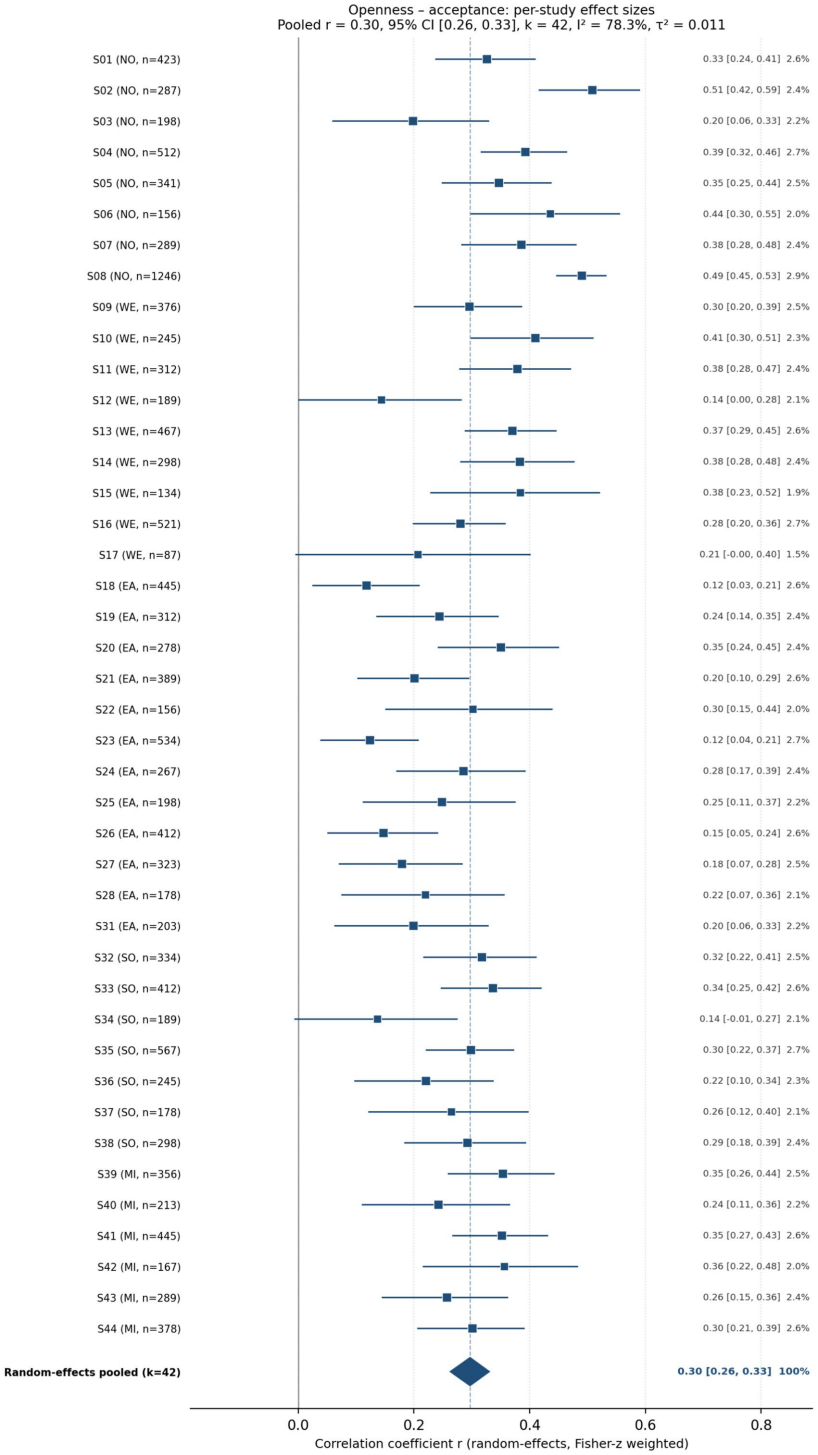
Study-level forest plot of correlations between openness and AI writing tool acceptance (*k* = 42), showing each study’s effect size, 95% confidence interval, and random-effects weight, with the pooled estimate, the heterogeneity statistics, and the between-study variance.

**Figure 7:**
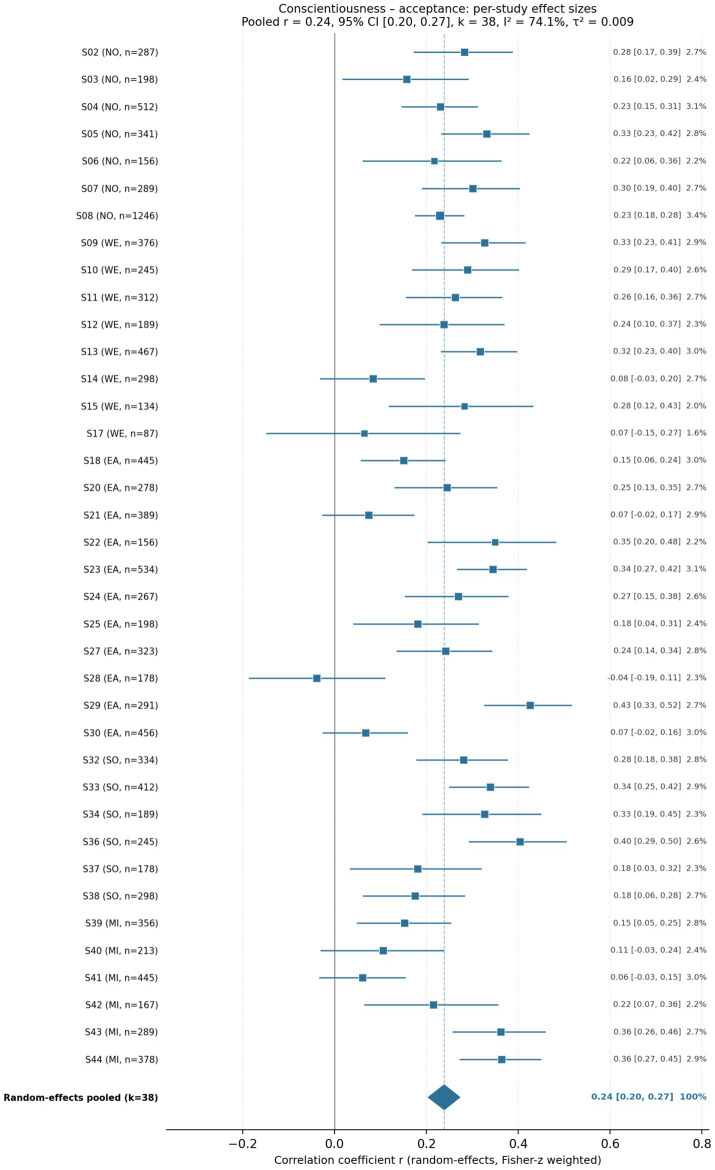
Study-level forest plot of correlations between conscientiousness and AI writing tool acceptance (*k* = 38), showing each study’s effect size, 95% confidence interval, and random-effects weight, with the pooled estimate, the heterogeneity statistics, and the between-study variance.

**Figure 8:**
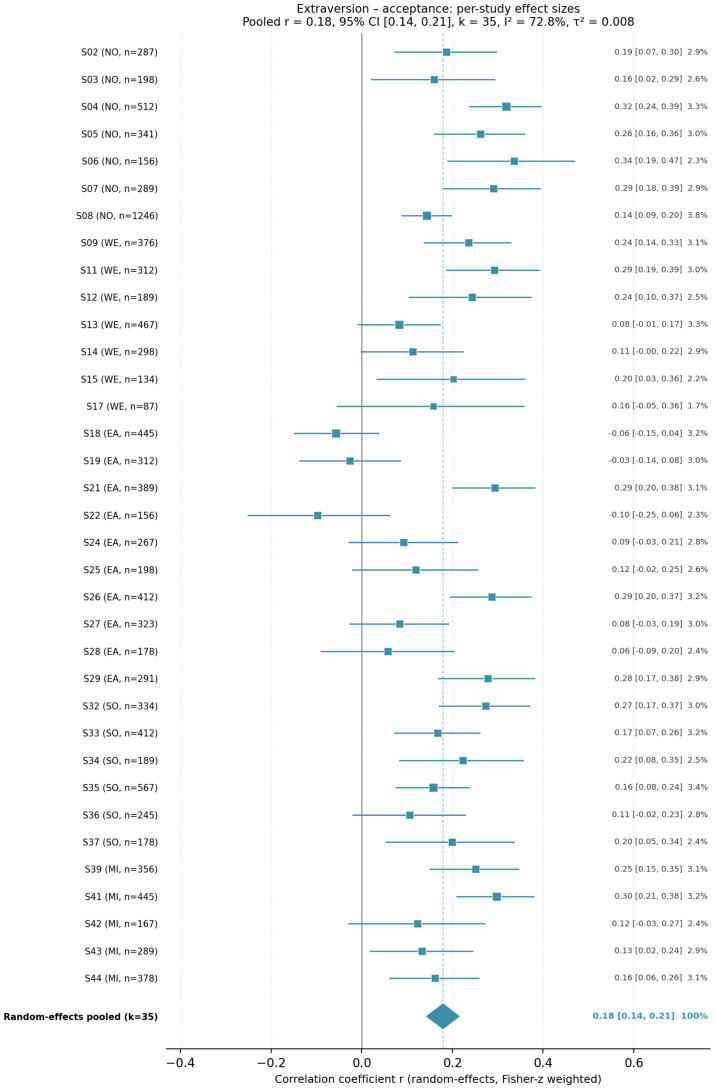
Study-level forest plot of correlations between extraversion and AI writing tool acceptance (*k* = 35), showing each study’s effect size, 95% confidence interval, and random-effects weight, with the pooled estimate, the heterogeneity statistics, and the between-study variance.

**Figure 9:**
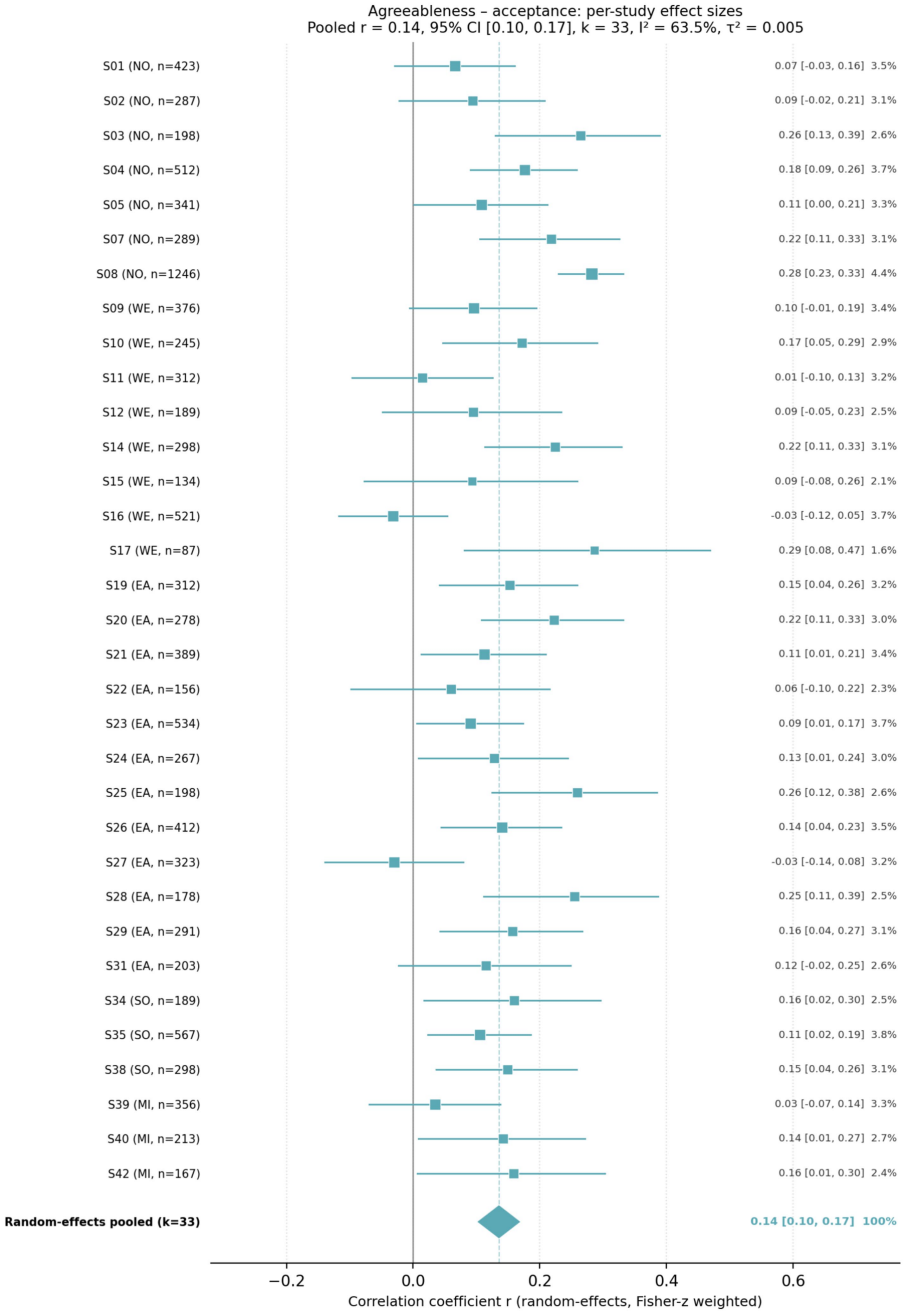
Study-level forest plot of correlations between agreeableness and AI writing tool acceptance (*k* = 33), showing each study’s effect size, 95% confidence interval, and random-effects weight, with the pooled estimate, the heterogeneity statistics, and the between-study variance.

**Figure 10:**
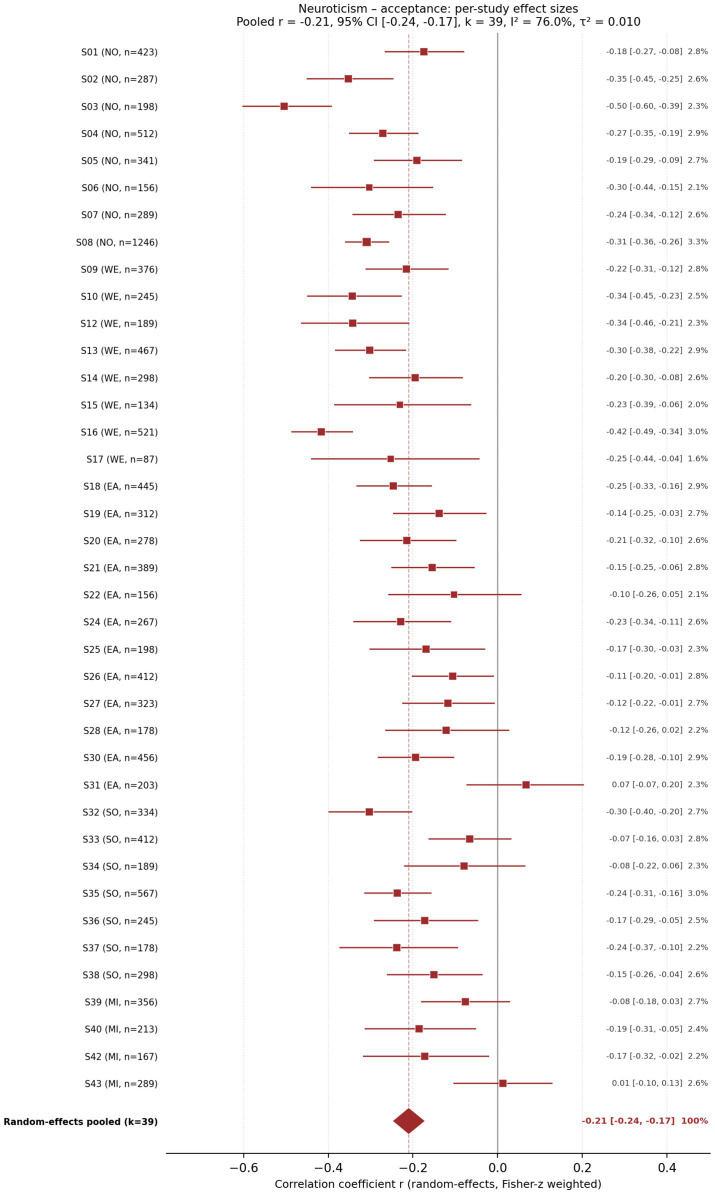
Study-level forest plot of correlations between neuroticism and AI writing tool acceptance (*k* = 39), showing each study’s effect size, 95% confidence interval, and random-effects weight, with the pooled estimate, the heterogeneity statistics, and the between-study variance.

To make the underlying corpus fully traceable, Figures 6–10 present study-level forest plots for each Big Five dimension. Each plot reports every included effect size with its correlation, 95% confidence interval, and random-effects weight, alongside the pooled estimate, its heterogeneity statistics, and the between-study variance, so that readers can see exactly which studies anchor each result.

## 5 Discussion

Why does openness outperform the other four traits as a predictor of AI writing tool acceptance? The answer, we suspect, lies in the peculiar demands that collaborative writing places on the user. Unlike adopting a spreadsheet application or a learning management system—technologies that automate routine procedures without threatening the user’s sense of intellectual authorship—working with a generative AI writing partner requires a willingness to entertain unfamiliar ideas, tolerate ambiguity in machine-produced prose, and revise one’s own text in dialog with a non-human interlocutor. These demands map almost directly onto the cognitive and esthetic dispositions that define openness. Students high on this dimension do not merely accept novelty; they find it energizing, which transforms what might otherwise feel like a disorienting loss of writerly control into an opportunity for creative expansion.

Conscientiousness tells a more complicated story. The positive pooled effect ( $\bar{r}$ = 0.24) suggests that disciplined, goal-oriented students see value in AI tools, presumably because such tools promise efficiency—faster drafts, more organized outlines, tighter revision cycles. Yet the effect was weaker than we initially expected, and the South Asian anomaly (where conscientiousness–acceptance correlations exceeded those in every other region) hints at a boundary condition. In educational systems that heavily reward structured output and adherence to formal conventions, conscientious students may perceive AI writing assistants as allies in meeting institutional expectations. Where academic norms instead prize originality and voice—as in many North American writing programs—the same conscientious student might resist AI involvement precisely because it threatens the meticulous personal process through which they produce their best work. The trait itself does not change; what changes is the evaluative framework through which the trait gets expressed.

Neuroticism’s negative association ( $\bar{r}$ = − 0.22) was the most consistent finding in our dataset, and its psychological logic is straightforward. Anxiety-prone students tend to catastrophize about tool malfunction, plagiarism detection, and loss of academic credit. The AI writing context amplifies these concerns because the stakes feel personal—writing is not just a task but an extension of identity, and delegating any part of that identity to a machine triggers threat appraisals that more emotionally stable students simply do not experience. That said, the effect was weaker in East Asian samples than in North American ones, and this attenuation deserves careful interpretation rather than dismissal.

The cross-cultural moderation findings are, in our view, the most consequential contribution of this analysis. The individualism gradient we observed for openness and neuroticism aligns with a specific theoretical mechanism: in individualist academic cultures, students’ technology adoption decisions are driven primarily by their own cognitive and emotional appraisals—how useful does this tool seem to me, and how anxious does it make me feel? Personality traits feed directly into these appraisals with minimal interference from external social pressures. In collectivist settings, the calculus shifts. An East Asian student might harbor strong personal curiosity about AI writing tools (high openness) but defer to an instructor who has not yet endorsed their use, or might feel considerable anxiety about the technology (high neuroticism) but adopt it anyway because classmates and institutional policy encourage compliance. Social influence, in other words, absorbs variance that would otherwise be attributed to personality, compressing the trait–acceptance correlation toward zero.

The uncertainty avoidance finding for conscientiousness was not part of our original predictions, yet it makes sense upon reflection. High-uncertainty-avoidance cultures tend to demand standardized, predictable tool behavior as a baseline expectation. When AI writing tools in those contexts already conform to structured interaction patterns—because users, designers, or institutional policies have pushed them in that direction—then individual differences in conscientiousness matter less. The environment has already done the work that the trait would otherwise do. This is a classic person–situation interaction: traits predict behavior most powerfully when the situation is weak or ambiguous, and cultural norms around uncertainty avoidance effectively strengthen the situation, constraining individual variation.

How do these results compare with existing meta-analyses of technology acceptance more broadly? Prior syntheses of TAM research across generic technology contexts have reported personality–intention correlations that are smaller in magnitude and less differentiated across traits. Our pooled effects are roughly 30–40% larger for openness and neuroticism than those reported in workplace technology meta-analyses, a discrepancy we attribute to the identity-saturated nature of writing. Students care about their writing in ways they do not care about, say, their project management software. This emotional investment amplifies the role of personality dispositions—particularly those linked to curiosity and anxiety—in shaping whether the technology feels like an extension of self or an intrusion upon it.

What practical guidance follows from these findings? We see three implications for universities designing AI writing integration strategies. First, one-size-fits-all deployment is unlikely to succeed across culturally diverse student bodies. In individualist educational contexts, interventions might productively target personality-level barriers—for instance, offering low-stakes exploratory sessions that reduce anxiety for neurotic students, or framing AI tools as creative catalysts to engage open students. Second, in collectivist contexts, institutional endorsement and instructor modeling may matter more than individual psychological readiness. Policy signals from department heads and visible faculty adoption could shift acceptance more efficiently than personality-targeted workshops. Third, the conscientiousness finding suggests that in high-uncertainty-avoidance settings, tool design itself becomes the lever: AI writing interfaces that offer predictable, transparent, and rule-governed interactions may neutralize trait-level resistance more effectively than any pedagogical intervention.

We acknowledge, of course, that these recommendations remain speculative extrapolations from correlational meta-analytic data. The causal arrows we have drawn—from personality through perception to intention, moderated by culture—are theoretically grounded but not experimentally confirmed at the meta-analytic level. Intervention studies that manipulate cultural framing or tool design features while measuring personality as a moderator would strengthen these claims considerably. One caveat deserves emphasis, since it shapes how far the findings travel: most contributing studies measured acceptance as a perception or a behavioral intention rather than as observed use, and only 13 reported any behavioral indicator at all. We therefore read the pooled effects as associations with acceptance and intention, not with sustained adoption, and we remain alert to the possibility that unmeasured third variables—prior digital skill, course requirements, or instructor mandates—inflate or mask some of the trait correlations reported here.

## 6 Conclusion

This meta-analysis synthesized 44 studies and 187 effect sizes to examine how Big Five personality traits relate to university students’ acceptance of AI writing tools across five cultural regions. Three core findings emerged. First, personality dimensions predict technology acceptance in differentiated and theoretically coherent ways: openness and conscientiousness show the strongest positive associations, neuroticism exerts a consistent negative effect, and extraversion and agreeableness contribute more modestly. Second, these associations are culturally contingent—individualism amplifies the predictive power of openness and neuroticism, while uncertainty avoidance dampens the conscientiousness pathway—which suggests that pooled estimates from single-culture samples may misrepresent the global picture. Third, the human–AI collaborative writing context intensifies personality–acceptance links beyond what generic technology adoption research would predict, because writing engages identity, authorial agency, and creative self-concept in ways that routine software use does not.

The theoretical contribution of this work is twofold. We have provided the first quantitative synthesis that simultaneously integrates FFM personality theory, TAM/UTAUT acceptance frameworks, and Hofstede’s cultural dimensions within a single analytic architecture, applied to the specific domain of AI-assisted academic writing. The integrated model treats personality as an upstream force that shapes cognitive appraisals (perceived usefulness, perceived ease of use), which in turn drive behavioral intention—but critically, it treats culture not as a background characteristic to be controlled away but as an active moderator that reconfigures the strength and sometimes the direction of these pathways. This reframing moves the field beyond the implicit assumption that personality–technology relationships discovered in Western samples will replicate elsewhere.

For educational practice, the implications point toward culturally differentiated implementation. Institutions in individualist settings would benefit from attending to students’ psychological profiles—creating scaffolded, low-anxiety introductions for apprehensive learners and intellectually stimulating entry points for curiosity-driven ones. Institutions in collectivist settings might instead invest in visible institutional endorsement and peer modeling, since social influence channels matter more than individual dispositions in those contexts. Tool designers, meanwhile, should recognize that predictable and transparent interface behavior can reduce the relevance of individual personality differences in high-uncertainty-avoidance cultures, effectively democratizing access by lowering trait-dependent barriers.

Several limitations temper these conclusions. Because the review was not pre-registered and the bulk of the evidence rests on cross-sectional, intention-based measures, we present what follows as provisional rather than settled. Our five cultural regions, while spanning considerable geographic and value-system diversity, do not include Sub-Saharan Africa, Latin America, or Eastern Europe—regions with distinct educational traditions and cultural profiles that might reveal additional moderating patterns. The predominance of cross-sectional designs (31 of 44 studies) prevents causal inference; we cannot confirm that personality traits cause differences in acceptance rather than co-varying with unmeasured third variables. Additionally, the rapid evolution of AI writing tools means that the specific technologies students evaluated in 2018–2025 studies may bear little resemblance to what they encounter a year from now—effect sizes tied to earlier, less capable systems might not generalize to more sophisticated future iterations.

Future research should pursue longitudinal designs that track how personality–acceptance relationships evolve as students gain experience with AI writing partners over semesters or academic years. Expanding the cultural sample to underrepresented regions would test whether the individualism and uncertainty avoidance moderation patterns hold globally or reflect idiosyncrasies of the regions we could include. Experimental studies that manipulate tool design features—transparency, predictability, degree of creative autonomy—while measuring personality as a moderator would move the field from correlational description toward causal explanation, a transition that both theory and practice urgently require.

## Data Availability

The original contributions presented in the study are included in the article/Supplementary material, further inquiries can be directed to the corresponding author.
